# Atherogenic index of plasma: a new indicator for assessing the short-term mortality of patients with acute decompensated heart failure

**DOI:** 10.3389/fendo.2024.1393644

**Published:** 2024-06-10

**Authors:** Meng Yu, Hongyi Yang, Maobin Kuang, Jiajun Qiu, Changhui Yu, Guobo Xie, Guotai Sheng, Yang Zou

**Affiliations:** ^1^Department of Cardiology, Jiangxi Provincial People’s Hospital, The First Affiliated Hospital of Nanchang Medical College, Nanchang, Jiangxi, China; ^2^Department of Ultrasound, the Second Affiliated Hospital of Nanchang University, Nanchang, Jiangxi, China; ^3^Jiangxi Medical College, Nanchang University, Nanchang, Jiangxi, China; ^4^Jiangxi Cardiovascular Research Institute, Jiangxi Provincial People’s Hospital, The First Affiliated Hospital of Nanchang Medical College, Nanchang, Jiangxi, China

**Keywords:** arteriosclerosis, atherogenic index of plasma, acute decompensated heart failure, poor prognoses, AIP, ADHF

## Abstract

**Objective:**

Arteriosclerosis is a primary causative factor in cardiovascular diseases. This study aims to explore the correlation between the atherogenic index of plasma (AIP) and the 30-day mortality rate in patients with acute decompensated heart failure (ADHF).

**Methods:**

A total of 1,248 ADHF patients recruited from the Jiangxi-Acute Decompensated Heart Failure1 (JX-ADHF1) cohort between 2019 and 2022 were selected for this study. The primary outcome was the 30-day mortality rate. Multivariable Cox regression, restricted cubic splines (RCS), and stratified analyses were utilized to assess the relationship between AIP and the 30-day mortality rate in ADHF patients. Mediation models were employed for exploratory analysis of the roles of inflammation, oxidative stress, and nutrition in the association between AIP and the 30-day mortality rate in ADHF patients.

**Results:**

During the 30-day follow-up, 42 (3.37%) of the ADHF patients died. The mortality rates corresponding to the quartiles of AIP were as follows: Q1: 1.28%, Q2: 2.88%, Q3: 2.88%, Q4: 6.41%. The multivariable Cox regression revealed a positive correlation between high AIP and the 30-day mortality rate in ADHF patients [Hazard ratio (HR) 3.94, 95% confidence interval (CI): 1.08–14.28], independent of age, gender, heart failure type, cardiac function classification, and comorbidities. It is important to note that there was a U-shaped curve association between AIP (<0.24) and the 30-day mortality rate before the fourth quartile, with the lowest 30-day mortality risk in ADHF patients around an AIP of -0.1. Furthermore, mediation analysis suggested significant mediating effects of inflammation and nutrition on the 30-day mortality rate in ADHF patients related to AIP, with inflammation accounting for approximately 24.29% and nutrition for about 8.16% of the mediation effect.

**Conclusion:**

This retrospective cohort analysis reveals for the first time the association between AIP and the 30-day mortality rate in ADHF patients. According to our findings, maintaining an AIP around -0.1 in ADHF patients could be crucial for improving poor prognoses from a medical perspective. Additionally, for ADHF patients with high AIP, it is important to assess and, if necessary, enhance nutritional support and anti-inflammatory treatment.

## Introduction

ADHF is characterized by new-onset or worsening symptoms and signs of severe cardiac functional or structural abnormalities, necessitating urgent hospitalization (1). Despite significant advancements in heart failure medications, assistive devices, and therapeutic approaches over the past few decades (2–7), the recurrent hospitalization and mortality risks remain notably high for ADHF patients (8–11). Therefore, early and effective risk stratification using simple factors could be crucial in improving the prognosis for hospitalized ADHF patients.

Arteriosclerosis is a major cause of various cardiovascular and cerebrovascular diseases such as coronary heart disease, heart failure, and stroke (12–14). Early identification of arteriosclerosis is vital in reducing the burden of these diseases (11–15). Clinical follow-up studies have confirmed the importance of assessing arteriosclerosis for risk stratification in heart failure, which includes using measures like carotid intima-media thickness as a marker of carotid arteriosclerosis (16, 17), the ankle-brachial index for peripheral artery disease (18, 19), and coronary artery calcification scoring as a sign of coronary arteriosclerosis (20, 21). The AIP is a novel and simple biological marker for assessing arteriosclerosis, developed by Professors Dobiásová M and Frohlich J, calculated from triglycerides (TG) and high-density lipoprotein cholesterol (HDL-C) levels (22). Dobiásová M and colleagues, in their evaluation across 35 cohorts with varying risks of arteriosclerosis, found that the AIP measurements closely matched the values of lipoprotein particle size and the fractional esterification rate of high-density lipoprotein cholesterol, and were directly related to arteriosclerosis risk. Hence, they recommended AIP as an assessment parameter for arteriosclerosis. This recommendation has been validated in numerous subsequent clinical cohorts (23–27), where researchers have highlighted the significance of AIP in assessing cardiovascular and cerebrovascular diseases and their adverse outcomes. However, the role of AIP in the prognosis of ADHF patients remains unclear. To address this question, our current study aims to analyze the association between AIP and the 30-day mortality rate in ADHF patients, exploring key mediating pathways that contribute to this association, through the JX-ADHF1 cohort.

## Methods

### Subject selection

**Figure 1:**
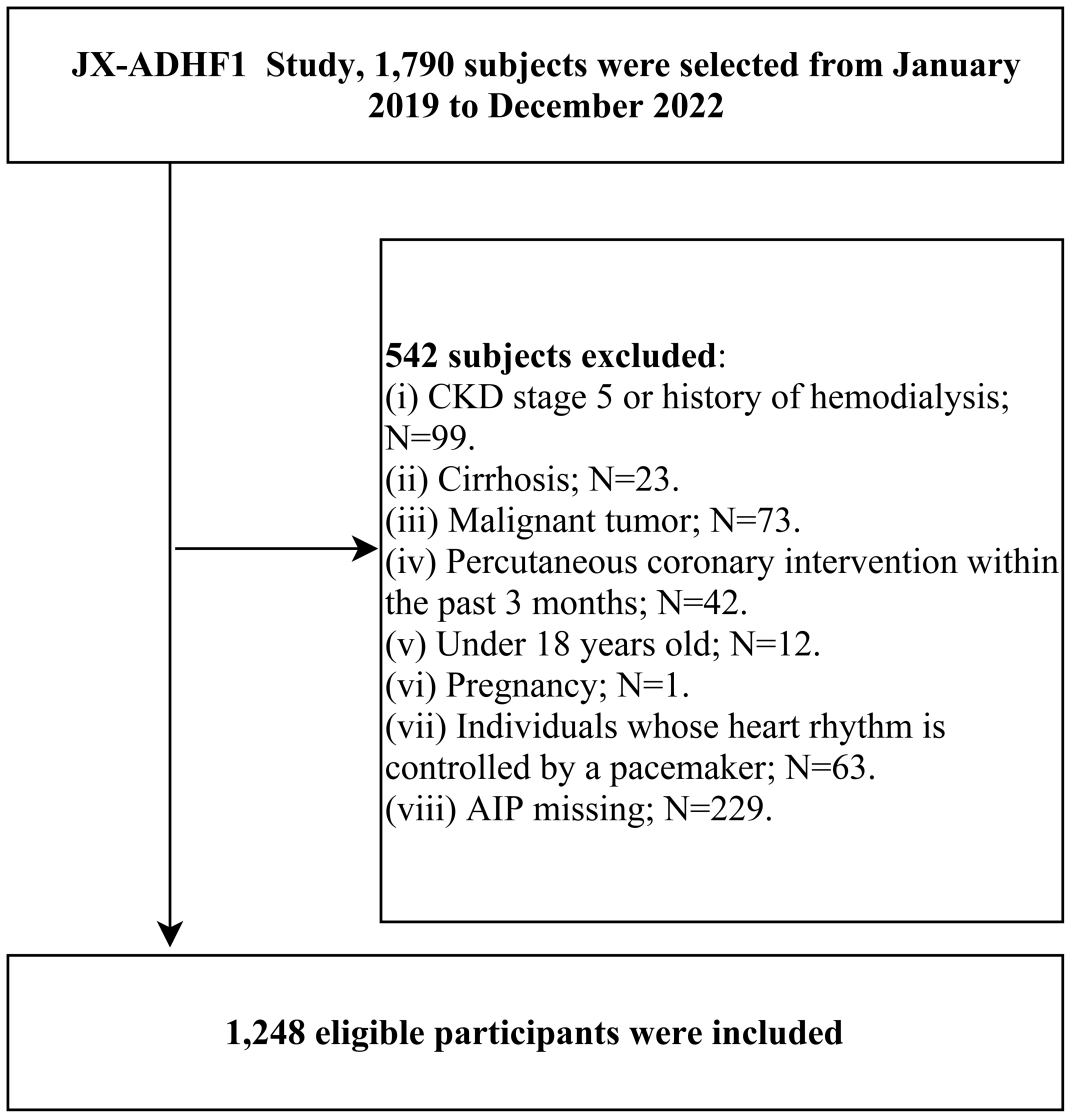
Flow chart of study participants.

The JX-ADHF1 study is a retrospective cohort study initiated by medical professionals. Its primary objective is to establish a high-quality cohort of ADHF patients, effectively utilizing clinical records during hospitalization to explore new methods for early risk stratification and improve the prognosis of ADHF patients. Specifically, the JX-ADHF1 study consecutively included 1,790 ADHF patients admitted to Jiangxi Provincial People’s Hospital from January 2019 to December 2022. The diagnosis of ADHF was based on the latest European Society of Cardiology guidelines for acute and chronic heart failure available at the time of admission. The exclusion criteria for the study population were as follows: (i) 23 subjects with liver cirrhosis and 99 subjects with stage 5 chronic kidney disease or a history of hemodialysis were excluded due to the potential adverse impact of non-heart failure related fluid retention on the study factors and prognosis. (ii) 42 subjects who underwent percutaneous coronary intervention within the last three months were excluded due to the significant impact of reperfusion therapy on short-term prognosis. (iii) 73 subjects with malignant tumors were excluded due to their potentially life-limiting prognosis impacting the study results. (iv) 63 patients with pacemakers were excluded due to expected autonomic regulatory deficits. (v) 12 minors and 1 pregnant woman were also excluded. Finally, for the current study, 229 subjects with missing baseline AIP information were excluded. Ultimately, the current study evaluated 1,248 ADHF patients, and **Figure 1** depicts the detailed screening process for the entire study population.

### Ethics and informed consent

The JX-ADHF1 study data, owned by Jiangxi Provincial People’s Hospital, is accessible to researchers who have signed a data use agreement. The JX-ADHF1 study protocol was approved by the Ethics Committee of Jiangxi Provincial People’s Hospital (IRB: 2024–01), and consent for data use was obtained from the subjects and their families. The research dataset anonymized personal identifiers with identifiable information, adhering to the ethical principles of the Declaration of Helsinki and reported results according to the STROBE guidelines **Supplementary Text 1**.

### Data collection

Demographic (gender and age) and clinical data [comorbidities (hypertension, diabetes, cerebral infarction, coronary heart disease), New York Heart Association (NYHA) classification at admission, systolic and diastolic blood pressure (SBP and DBP), echocardiogram results at admission, and blood sample laboratory parameters] of the subjects at baseline were independently collected and cross-checked by two trained researchers. The blood pressure measurements recorded in the current analysis were the first measurements after admission, taken in a calm environment or bedside using an Omron automatic sphygmomanometer (HBP-1300). Comorbidities were determined based on patient self-report, ongoing medication treatment, or records in the patient’s medical history.

Laboratory parameters were measured within 24 hours of admission at the Jiangxi Provincial People’s Hospital laboratory center by professional medical laboratory personnel using automatic analyzers. The biochemical indicators measured included albumin, alanine aminotransferase (ALT), aspartate aminotransferase (AST), gamma-glutamyl transferase (GGT), creatinine (Cr), uric acid (UA), total cholesterol (TC), TG, low-density lipoprotein cholesterol (LDL-C), and HDL-C, routine blood parameters [white blood cell count (WBC), red blood cell count (RBC), hemoglobin (HGB), platelet count (PLT)], and the cardiac function indicator N-terminal pro B-type natriuretic peptide (NT-proBNP). It is important to note that lipid and liver enzyme-related indicators were measured from venous blood samples taken on an empty stomach at admission or the next morning after admission.

### Calculation of AIP

AIP = log10 (TG/HDL-C) (22).

### Study outcome

The primary outcome of the study is the all-cause 30-day mortality rate. The start of the follow-up for all ADHF patients is set at the time of admission, and their 30-day survival status is obtained by trained medical workers through text messages, phone calls, and face-to-face follow-ups in outpatient and inpatient settings.

### Statistical analysis

Data analysis in this study is performed using R language version 4.2.1 and Empower(R) version 2.20 statistical software. Baseline characteristics of the study population were described as counts (%), mean (standard deviation), or median (interquartile range) based on the type and distribution of variables. Differences between groups were compared using t-tests, one-way ANOVA, and non-parametric tests, with a two-sided *P* < 0.05 set as the threshold for statistical significance.

Kaplan-Meier curve was used to depict the 30-day survival rates of ADHF patients. Cox regression models were constructed to test the association between AIP and the 30-day mortality rate in ADHF patients, in which the variance inflation factors of all covariates were considered in the adjustment of variables (28), and evaluated the suitability of the Cox regression model using Schoenfeld residuals to assess the proportional hazards assumption (29). To test the robustness of the Cox regression analysis, the minimum strength of association needed for an unmeasured confounder to explain the 30-day mortality rate in ADHF patients was calculated based on the final adjusted model (E-value) (30).

Nested within the Cox regression model, RCS with four knots was employed to model the dose-response relationship between AIP and the 30-day mortality rate in ADHF patients. Stratified analysis was further used to explore how the association between AIP and the 30-day mortality rate in ADHF patients varies across different subgroups, with likelihood ratio tests assessing differences between strata.

After establishing the association between AIP and the 30-day mortality rate in ADHF patients, mediation analysis (31) was conducted to explore whether oxidative stress (32), inflammation (33), and nutrition (34) pathways mediate the relationship between AIP and the 30-day mortality rate. The size of the mediation effect was quantified by calculating the ratio of the indirect effect to the total effect, and the significance of the mediation effect was tested using the Bootstrap sampling method (35). Based on previous studies, GGT is selected as a marker of oxidative stress (36), WBC as a marker of inflammation (37), and ALB as an indicator of nutritional status (38).

## Results

### Follow-up outcomes and baseline characteristics

**Figure 2:**
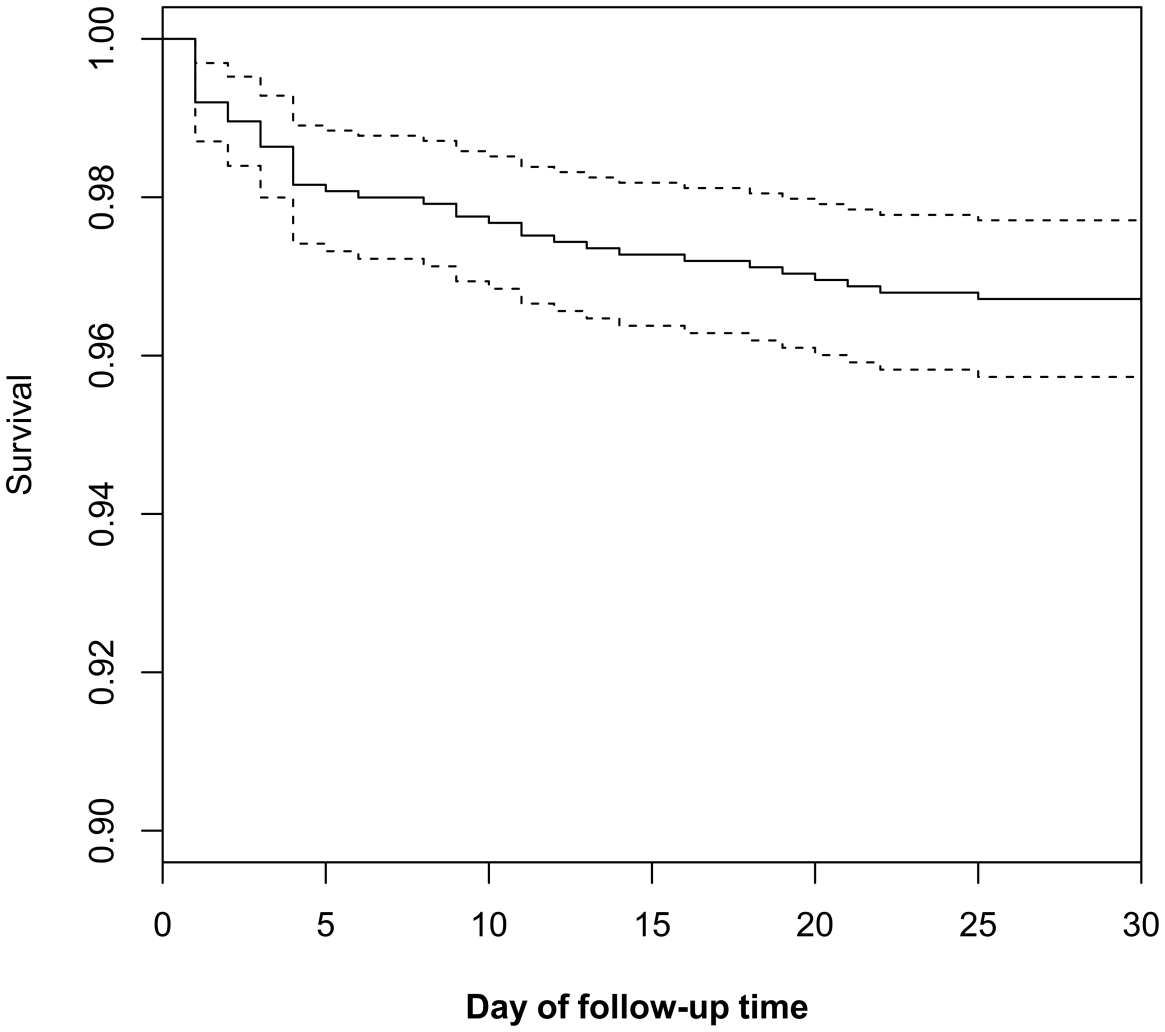
30-day survival curve of acute decompensated heart failure patients.

The analysis included 1,248 ADHF patients with an average age of 68 years and a male-to-female ratio of 1.43:1. During the 30-day observation period, 42 patients (3.37%) experienced mortality events. **Figure 2** displays the 30-day survival curve of the study population, indicating a gradual increase in mortality events over time within the 30-day period.

In this analysis, AIP in ADHF patients showed a normal distribution (**Supplementary Figure 1**), with a median of 0.075. Patients were divided into quartiles based on their AIP, summarizing the baseline characteristics of ADHF patients (**Table 1**). The results indicated that compared to the group with lower AIP values, those with higher AIP were younger, had lower SBP, left ventricular ejection fraction (LVEF), ALB, and HDL-C levels, and higher levels of WBC, RBC, PLT, ALT, Cr, UA, TC, TG, and LDL-C. Additionally, patients with higher AIP were more likely to have comorbid diabetes and coronary heart disease, a higher proportion of NYHA class IV, and a significantly higher probability of mortality within 30 days (Q1: 1.28%, Q2: 2.88%, Q3: 2.88%, Q4: 6.41%).

**Table 1:** Summary of baseline characteristics of the study population according to AIP quartile group.

	AIP quartiles				*P*-value
	Q1 (-0.73–0.08)	Q2 (-0.08–0.08)	Q3 (0.08–0.24)	Q4 (0.24–1.55)	
No. of subjects	312	312	312	312	
Gender					0.709
Female	129 (41.35%)	134 (42.95%)	130 (41.67%)	120 (38.46%)	
Male	183 (58.65%)	178 (57.05%)	182 (58.33%)	192 (61.54%)	
Age (years)	74.00 (67.00–81.00)	70.00 (60.00–80.00)	69.00 (60.00–78.00)	66.00 (57.00–75.00)	<0.001
Comorbidities					
Hypertension (n,%)	128 (41.03%)	122 (39.10%)	137 (43.91%)	141 (45.19%)	0.405
Diabetes (n,%)	37 (11.86%)	87 (27.88%)	83 (26.60%)	121 (38.78%)	<0.001
Cerebral infarction (n,%)	58 (18.59%)	55 (17.63%)	43 (13.78%)	52 (16.67%)	0.406
CHD (n,%)	82 (26.28%)	98 (31.41%)	92 (29.49%)	123 (39.42%)	0.004
NYHA classification (n,%)					0.310
III	229 (73.40%)	222 (71.15%)	217 (69.55%)	208 (66.67%)	
IV	83 (26.60%)	90 (28.85%)	95 (30.45%)	104 (33.33%)	
SBP (mmHg)	130.80 (25.05)	128.95 (23.47)	127.64 (25.27)	125.53 (24.39)	0.055
DBP (mmHg)	76.70 (15.77)	76.18 (15.09)	76.16 (15.81)	75.20 (16.09)	0.684
LVEF (%)	48.00 (40.00–56.00)	48.00 (39.00–57.00)	46.00 (37.00–55.00)	45.00 (36.00–55.25)	0.026
WBC (×10^9^/L)	5.47 (4.49–6.90)	5.80 (4.70–7.48)	6.37 (5.26–7.96)	6.80 (5.60–8.71)	<0.001
RBC (×10^12^/L)	3.96 (0.69)	4.05 (0.72)	4.16 (0.75)	4.19 (0.88)	<0.001
HGB (g/L)	120.07 (20.66)	122.82 (22.13)	125.50 (22.28)	124.86 (25.37)	0.013
PLT (×10^9^/L)	148.00 (115.50–187.00)	161.00 (122.00–202.00)	166.00 (129.00–215.50)	177.50 (136.00–233.75)	<0.001
ALB (g/L)	35.92 (4.33)	35.57 (5.14)	35.37 (4.69)	34.70 (5.67)	0.019
ALT (U/L)	18.00 (12.00–29.75)	22.00 (14.00–35.25)	23.00 (14.00–40.00)	24.50 (14.75–45.00)	<0.001
AST (U/L)	25.00 (19.00–36.00)	25.00 (20.00–36.00)	25.00 (19.00–38.75)	25.00 (19.00–43.25)	0.619
GGT (U/L)	38.50 (22.25–64.25)	44.00 (25.00–83.00)	43.00 (25.00–75.75)	42.50 (25.00–87.25)	0.053
Cr (umol/L)	79.00 (61.50–102.00)	87.00 (66.00–121.00)	90.00 (72.00–120.00)	100.00 (73.00–165.00)	<0.001
UA (umol/L)	404.00 (329.00–504.00)	413.00 (334.50–518.75)	440.00 (361.00–555.00)	465.00 (346.00–606.00)	<0.001
TC (mmol/L)	3.72 (0.91)	3.79 (1.01)	3.77 (1.01)	3.96 (1.24)	0.026
TG (mmol/L)	0.77 (0.65–0.89)	1.06 (0.92–1.21)	1.29 (1.11–1.51)	1.89 (1.55–2.41)	<0.001
HDL-C (mmol/L)	1.26 (0.28)	1.07 (0.23)	0.93 (0.21)	0.77 (0.21)	<0.001
LDL-C (mmol/L)	2.14 (0.76)	2.36 (0.84)	2.45 (0.86)	2.57 (0.96)	<0.001
NT-proBNP (pmol/L)	3769.50 (2413.25–5707.25)	3500.50 (1917.50–5621.00)	3637.50 (2234.75–5519.75)	3875.00 (1971.75–5932.50)	0.468
30-day mortality	4 (1.28%)	9 (2.88%)	9 (2.88%)	20 (6.41%)	0.004

CHD, coronary heart disease; NYHA, New York Heart Association; LVEF, left ventricular ejection fraction; SBP, systolic blood pressure; DBP, diastolic blood pressure; TG, triglyceride; TC, total cholesterol; HDL-C, high-density lipoprotein cholesterol; LDL-C, low-density lipid cholesterol; Cr, creatinine; UA, uric acid; WBC, white blood cell count; RBC, red blood cell count; HGB, hemoglobin; PLT, platelet count; ALT, alanine aminotransferase; AST, aspartate aminotransferase; ALB, albumin; NT-proBNP, N-Terminal Pro-Brain Natriuretic Peptide; AIP, atherogenic index of plasma.

**Figure 3:**
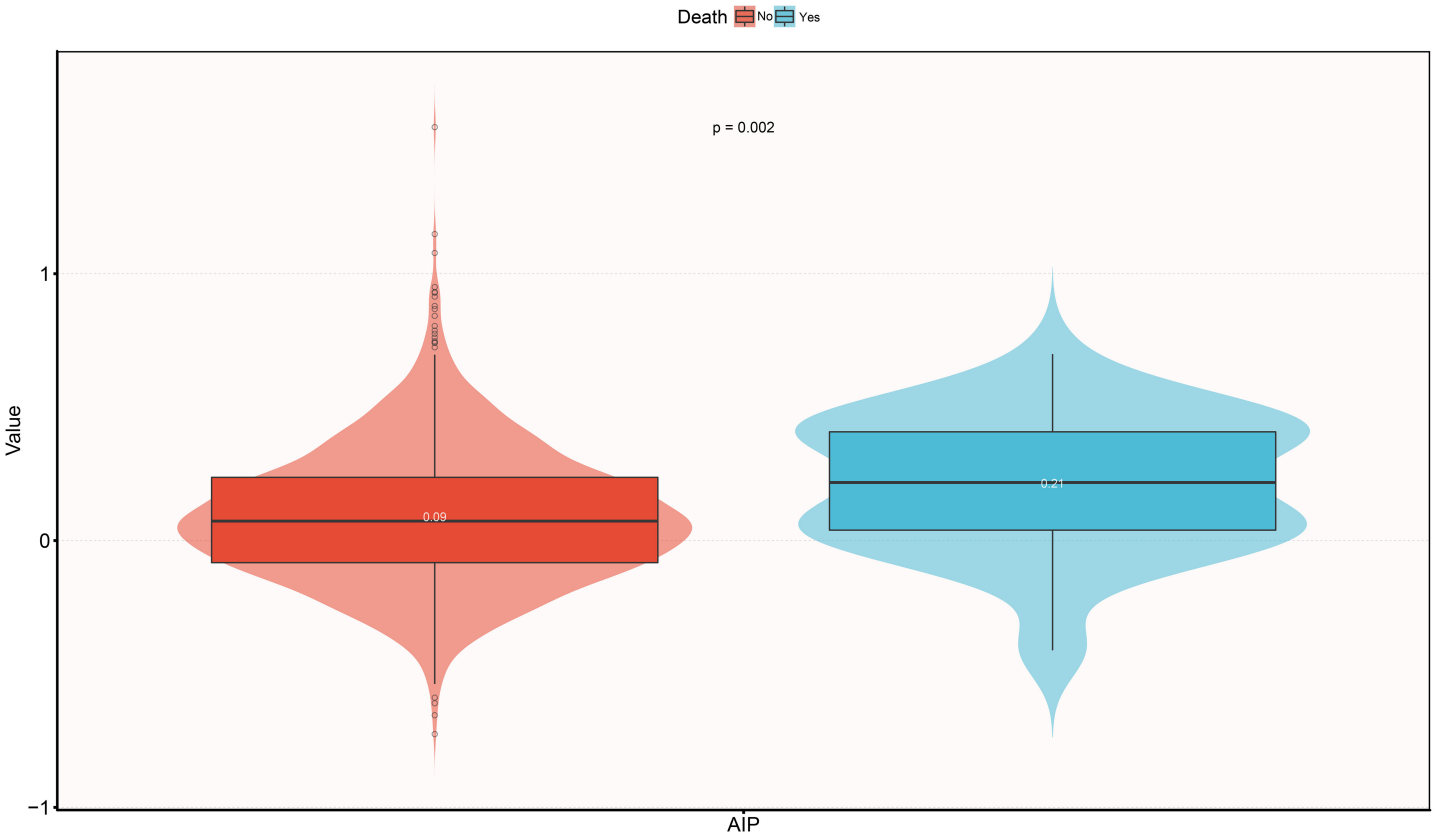
Violin diagram showing baseline characteristics of AIP according to whether death occurred during the 30-day follow-up period. AIP, atherogenic index of plasma.

**Table 2** further displays the baseline characteristics of the study population based on whether or not the subject experienced mortality within the 30-day follow-up period. In summary: (i) In demographic characteristics, ADHF patients who died within 30 days were significantly older at admission, had a higher proportion of NYHA class IV, and a higher prevalence of cerebral infarction. (ii) In terms of measured data, deceased subjects typically had lower baseline levels of blood pressure, RBC, HGB, PLT, ALB, TC, HDL-C, and LDL-C, but higher levels of WBC, liver enzymes, Cr, UA, TG, NT-proBNP, and AIP (**Figure 3**).

**Table 2:** Characteristics of study subjects surviving versus dying by 30 days.

	Survivors	Nonsurvivors	*P*-value
No. of subjects	1206	42	
Gender			0.470
Female	498 (41.29%)	15 (35.71%)	
Male	708 (58.71%)	27 (64.29%)	
Age (years)	70.00 (60.00–78.75)	77.50 (69.00–83.50)	0.001
Comorbidities			
Hypertension (n,%)	509 (42.21%)	19 (45.24%)	0.696
Diabetes (n,%)	313 (25.95%)	15 (35.71%)	0.158
Cerebral infarction (n,%)	194 (16.09%)	14 (33.33%)	0.003
CHD (n,%)	381 (31.59%)	14 (33.33%)	0.811
NYHA classification (n,%)			<0.001
III	863 (71.56%)	13 (30.95%)	
IV	343 (28.44%)	29 (69.05%)	
SBP (mmHg)	128.75 (24.41)	113.43 (25.80)	<0.001
DBP (mmHg)	76.42 (15.63)	65.67 (13.52)	<0.001
LVEF (%)	47.00 (38.00–56.00)	50.00 (38.50–53.00)	0.627
WBC (×10^9^/L)	6.10 (4.83–7.69)	10.21 (6.29–14.76)	<0.001
RBC (×10^12^/L)	4.10 (0.76)	3.72 (0.84)	0.001
HGB (g/L)	123.62 (22.69)	114.69 (23.06)	0.012
PLT (×10^9^/L)	161.00 (126.00–210.00)	151.50 (98.00–232.25)	0.499
ALB (g/L)	35.52 (4.92)	31.72 (5.63)	<0.001
ALT (U/L)	21.00 (13.00–37.00)	29.00 (15.00–81.50)	0.034
AST (U/L)	25.00 (19.00–37.00)	41.00 (22.00–98.50)	<0.001
GGT (U/L)	41.00 (24.00–76.00)	57.00 (30.00–93.75)	0.049
Cr (umol/L)	87.00 (67.00–120.00)	179.50 (124.00–276.75)	<0.001
UA (umol/L)	426.00 (342.00–539.00)	523.50 (391.25–661.00)	0.003
TC (mmol/L)	3.83 (1.05)	3.34 (0.83)	0.003
TG (mmol/L)	1.14 (0.88–1.55)	1.30 (1.03–1.77)	0.128
HDL-C (mmol/L)	1.01 (0.29)	0.85 (0.28)	<0.001
LDL-C (mmol/L)	2.39 (0.87)	2.01 (0.64)	0.005
AIP	0.09 (0.26)	0.21 (0.25)	0.002
NT-proBNP (pmol/L)	3618.50 (2140.25–5595.50)	6175.00 (3465.75–10123.00)	<0.001

CHD, coronary heart disease; NYHA, New York Heart Association; LVEF, left ventricular ejection fraction; SBP, systolic blood pressure; DBP, diastolic blood pressure; TG, triglyceride; TC, total cholesterol; HDL-C, high-density lipoprotein cholesterol; LDL-C, low-density lipid cholesterol; Cr, creatinine; UA, uric acid; WBC, white blood cell count; RBC, red blood cell count; HGB, hemoglobin; PLT, platelet count; ALT, alanine aminotransferase; AST, aspartate aminotransferase; ALB, albumin; NT-proBNP, N-Terminal Pro-Brain Natriuretic Peptide; AIP, atherogenic index of plasma.

### Association analysis of AIP with 30-day mortality in ADHF patients

Before analyzing the association, we confirmed through Schoenfeld residual plots of AIP over time (**Supplementary Figure 2**) that the Cox regression model did not violate the proportional hazards assumption. Additionally, the variance inflation factors of each covariate were calculated, identifying collinearity between HGB, ALB, and TC with other covariates, and thus these were not included in the subsequent multivariable models (**Supplementary Table 1**).

Three progressively adjusted multivariable Cox regression models were constructed to analyze the association between AIP and the 30-day mortality rate in ADHF patients (**Table 3**). In the first model (Model 1), adjustments were made for age, gender, and comorbidities (hypertension, diabetes, cerebral infarction, and coronary heart disease), showing a positive correlation between AIP and 30-day mortality rate in ADHF patients with an HR of 7.38 (95%CI: 2.55–21.36). Additionally, the HR values corresponding to the AIP quartiles showed a positive trend (*P*-trend=0.0002). In the second model (Model 2), adjustments were further made for NYHA classification, LVEF, DBP, and NT-proBNP, slightly decreasing the HR to 5.87 (95%CI: 1.77–19.44). In Model 2, despite a continuing positive trend in AIP quartiles, the HR value for the second quartile was slightly higher than for the third (HR: Q2 2.38, Q3 2.15). The third model (Model 3), further considered the impact of WBC, RBC, PLT, ALB, AST, GGT, Cr, UA, and LDL-C, with findings similar to Model 2, showing a slight decrease in HR (HR 3.94, 95%CI: 1.08–14.28). Notably, in Model 3, the HR value for the second quartile was higher than the third but lower than the fourth (HR: Q2: 2.82, Q3: 2.01, Q4: 4.09); suggesting a potential non-linear association before the fourth quartile of AIP. Based on Model 3, the minimum E-value associated with the 30-day mortality rate in ADHF patients was calculated to be 7.34.

**Table 3:** Multivariable Cox regression analysis of the association between AIP and 30-day mortality in patients with ADHF.

	Hazard ratios (95% confidence interval)		
	Model 1	Model 2	Model 3
AIP (continuous variable)	7.38 (2.55, 21.36)	5.87 (1.77, 19.44)	3.94 (1.08, 14.28)
AIP (quartiles)			
Q1(-0.73**–**0.08)	Ref	Ref	Ref
Q2(-0.08–0.08)	2.55 (0.78, 8.36)	2.38 (0.69, 8.15)	2.82 (0.78, 10.15)
Q3(0.08–0.24)	2.81 (0.86, 9.20)	2.15 (0.64, 7.28)	2.01 (0.51, 7.89)
Q4(0.24–1.55)	6.98 (2.31, 21.05)	4.89 (1.60, 14.96)	4.09 (1.25, 13.35)
*P*-trend	0.0002	0.0035	0.0233

AIP, atherogenic index of plasma; ADHF, acute decompensated heart failure.

Model 1 adjusted for gender, age, hypertension, diabetes, cerebral infarction and CHD.

Model 2 adjusted for model 1 + NYHA classification, LVEF, DBP, NT-proBNP.

Model 3 adjusted for: Model 2+ WBC, RBC, PLT, ALB, AST, GGT, Cr, UA, LDL-C.

### Dose-response relationship

**Figure 4:**
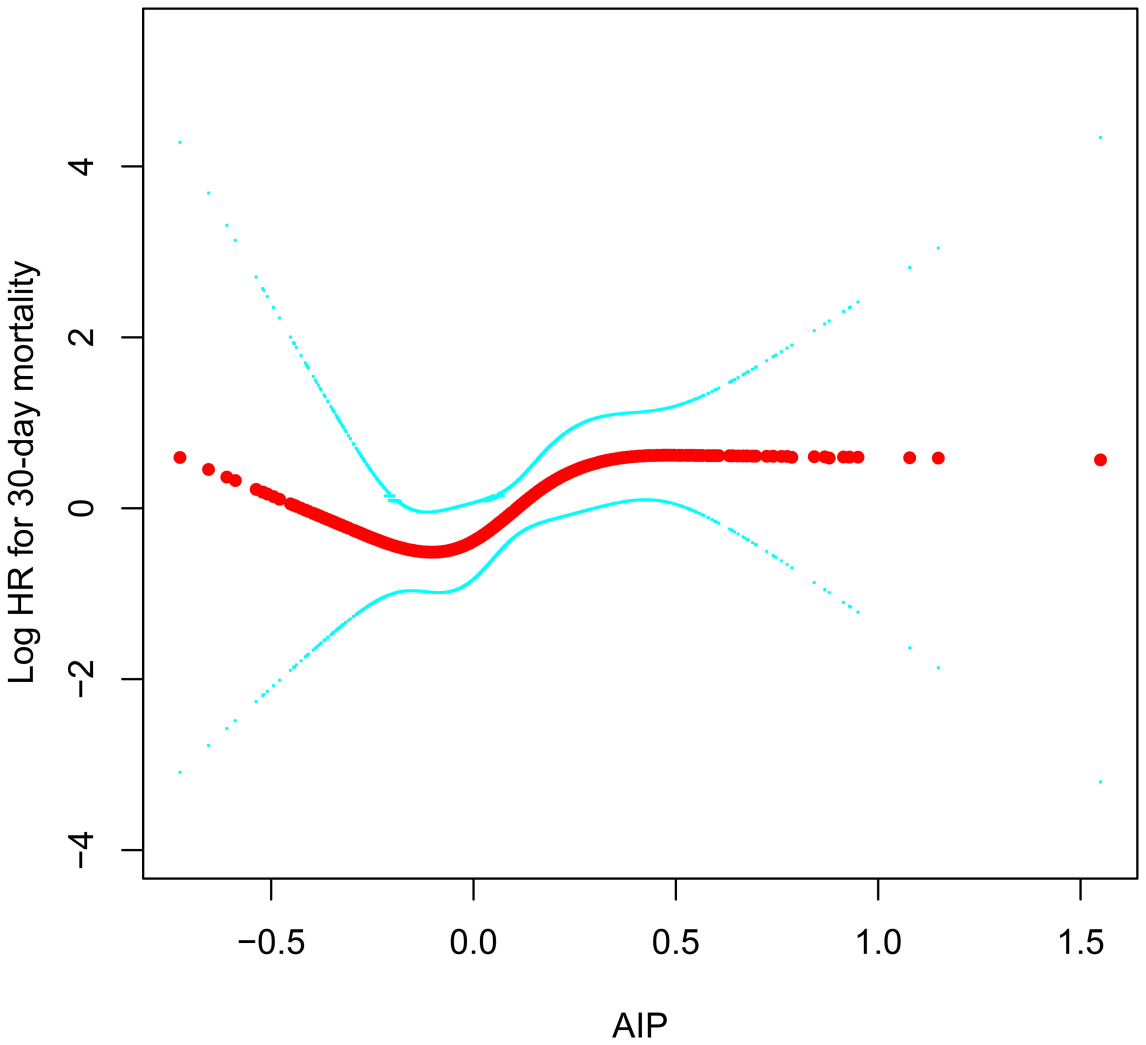
Fitting the dose-response relationship between AIP and 30-day mortality in ADHF patients with 4 knots restricted cubic spline. AIP, atherogenic index of plasma; ADHF, acute decompensated heart failure. Adjusted for gender, age, hypertension, diabetes, cerebral infarction, CHD, NYHA classification, LVEF, DBP, NT-proBNP, WBC, RBC, PLT, ALB, AST, GGT, Cr, UA, LDL-C.

Using a RCS with four knots, we further constructed a dose-response relationship curve between AIP in ADHF patients and their 30-day mortality rate (**Figure 4**). After adequately adjusting for confounding factors, we found that the dose-response curve correlated with the association analysis results in **Table 3**. It can be observed that the association between the 30-day mortality rate and AIP demonstrated a U-shaped curve before the fourth quartile of AIP (AIP<0.24), with the lowest 30-day mortality risk in ADHF patients occurring at an AIP of approximately -0.1.

### Subgroup analysis

We conducted stratified analyses based on age (median), gender, LVEF value (50%), NYHA classification, and comorbidities, assessing the presence of interactions between AIP and stratification factors through likelihood ratio tests. The results (**Table 4**) indicated no significant interactions in all the subgroups (All *P*-interaction>0.05), suggesting that the association between AIP and the 30-day mortality rate in ADHF patients was relatively stable and unlikely to be influenced by these external factors.

**Table 4:** Stratified analysis showed the relationship between AIP and 30-day mortality in patients with ADHF in different age, gender, NYHA class, LVEF and whether combined with hypertension/diabetes/cerebral infarction/CHD.

Subgroup	Adjusted HR (95%CI)	*P* for interaction
Age (years)		0.8800
20–70	6.24 (0.76, 51.04)	
71–96	7.70 (1.38, 42.87)	
Gender		0.8070
Male	4.37 (0.94, 20.19)	
Female	3.13 (0.33, 29.47)	
NYHA		0.9313
III	2.19 (0.16, 29.60)	
IV	2.50 (0.49, 12.67)	
LVEF		0.7670
< 50%	4.51 (0.66, 30.75)	
≥ 50%	3.09 (0.57, 16.80)	
Hypertension		0.9755
Yes	4.03 (0.54, 29.84)	
No	3.87 (0.73, 20.55)	
Diabetes		0.6974
Yes	2.64 (0.24, 29.15)	
No	4.65 (1.00, 21.54)	
Cerebral infarction		0.6899
Yes	5.91 (0.55, 63.24)	
No	3.37 (0.75, 15.16)	
CHD		0.0617
Yes	0.97 (0.13, 7.22)	
No	11.33 (2.22, 57.89)	

AIP, atherogenic index of plasma; ADHF, acute decompensated heart failure; CHD, coronary heart disease.

Models adjusted for the same covariates as in model 3 (**Table 3**), except for the stratification variable.

### Mediation analysis

**Figure 5:**
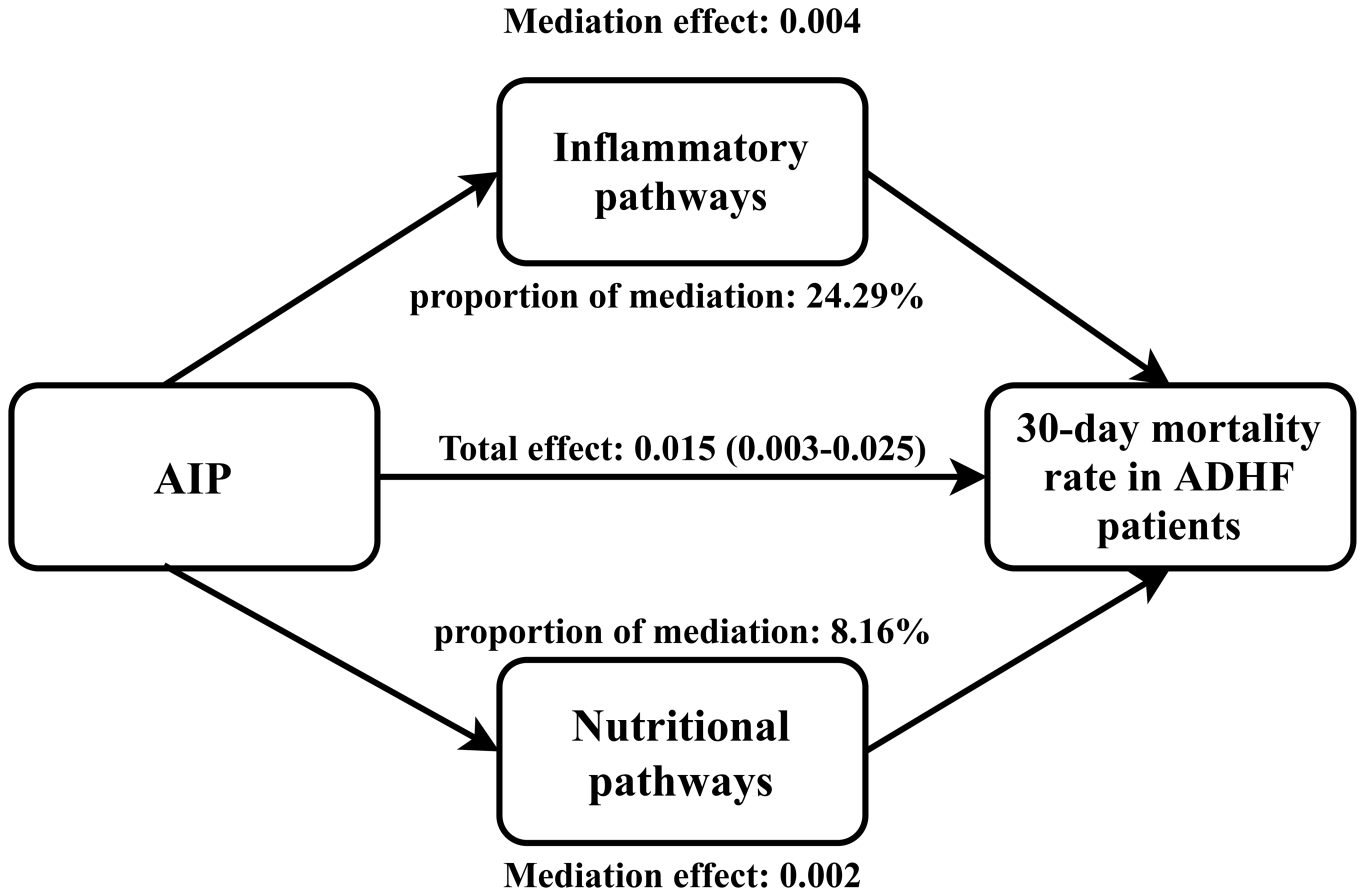
Path diagram for mediational model. AIP, atherogenic index of plasma; ADHF, acute decompensated heart failure. Adjusted for gender, age, hypertension, diabetes, cerebral infarction, CHD, NYHA classification, LVEF, DBP and NT-proBNP.

Mediated analysis was performed to explore the roles of inflammation, oxidative stress and nutritional pathways in the association between AIP and the 30-day mortality rate in ADHF patients. **Table 5** presents the detailed results of the mediation analysis, and **Figure 5** illustrates the mediation diagram for inflammation (WBC), oxidative stress (GGT), and nutrition (ALB). The exploratory analysis revealed significant mediating effects of inflammation and nutrition in the association between AIP and the 30-day mortality rate in ADHF patients (*P*-value of proportion mediate < 0.05), while the mediating effect of oxidative stress appeared non-significant (*P*-value of proportion mediate > 0.05). Specifically, inflammation accounted for approximately 24.29% of the mediation effect, and nutrition for about 8.16% in the association between AIP and the 30-day mortality rate in ADHF patients.

**Table 5:** Mediated analysis was performed to explore the roles of inflammation, oxidative stress and nutritional pathways in the association between AIP and the 30-day mortality rate in ADHF patients.

Mediator	Total effect	Mediation effect	Direct effect	PM(%)	*P*-value of PM
WBC	0.015 (0.003, 0.025)	0.004 (0.002, 0.006)	0.011 (0.002, 0.022)	24.29	0.032
GGT	0.015 (0.003, 0.025)	-0.000(-0.000,0.001)	0.015 (0.004, 0.026)	1.51	0.478
ALB	0.015 (0.003, 0.025)	0.002 (0.000, 0.003)	0.013 (0.002, 0.024)	8.16	0.042

PM, proportion mediate; ADHF, acute decompensated heart failure; other abbreviations as in **Table ​1**.

Model adjusted for the same covariates as in model 2 (**Table 3**), except for the mediator variable.

## Discussion

In this retrospective cohort analysis, we discovered that although the overall association between AIP at admission and 30-day mortality in patients with ADHF was positively correlated and independent of age, gender, heart failure type, cardiac function classification, and comorbidities, further analysis using RCS revealed a U-shaped association before the fourth quartile of AIP (AIP<0.24). The lowest 30-day mortality risk in ADHF patients corresponded to an AIP of approximately -0.1.

ADHF is a common reason for hospitalization or the need for emergency care in the elderly population and is associated with a high risk of adverse outcomes (1, 8–11). Statistics indicate that over 30% of ADHF patients require rehospitalization or face mortality shortly after discharge (within 90 days) (8–11, 39). In China, the 30-day mortality rate for ADHF patients ranges between 1.65% and 13.17% (40–45), while in the JX-ADHF1 cohort, it is approximately 4%, and in the current analysis, it is 3.37% after excluding certain subjects. ADHF has become one of the most challenging cardiovascular diseases to manage appropriately worldwide. Early identification of patients potentially at risk of adverse outcomes and exploration of more effective treatment options may be vital in reducing the disease burden on patients and the healthcare system (38).

AIP is a crucial assessment tool for arteriosclerosis. Many past studies have demonstrated its significant value in assessing the risk of cardiovascular and cerebrovascular diseases (23–26), and in evaluating adverse outcomes in these diseases (46–52). For instance, in assessing adverse outcomes in coronary heart disease patients, an increase of one unit in AIP as a continuous variable is reported to increase the risk of major adverse cardiovascular and cerebrovascular events by 30.8%-114.9% (46, 47). When AIP is considered as a categorical variable, patients with coronary heart disease and high AIP face a 61.4%-689% increased risk of major adverse cardiovascular and cerebrovascular events compared to those with low AIP (27, 48, 49). Overall, high AIP in patients with coronary heart disease indicates a higher risk of adverse outcomes. Similarly, in patients with baseline hypertension and stroke, high AIP also indicates a high risk of severe adverse outcomes (50–52). Our current study further revealed the role of AIP in assessing the risk of adverse outcomes in ADHF patients. The study showed that for each unit increase in AIP as a continuous variable, the 30-day mortality risk in ADHF patients increased by 294%; compared to those with low AIP, patients with high AIP had a 309% increased risk of 30-day mortality. These new research findings further underscored the significant value of AIP in assessing the risk of adverse outcomes in cardiovascular and cerebrovascular diseases.

An important finding of our current study, derived from the RCS analysis, is the U-shaped association between AIP and the 30-day mortality rate in ADHF patients before the fourth quartile of AIP (<0.24), with the lowest risk of 30-day mortality around an AIP of -0.1. This observation conveyed two crucial messages: (i) The dose-response curve provided a more intuitive understanding of the overall and stage-specific associations between AIP and the 30-day mortality rate in ADHF patients. (ii) The nadir of the U-shaped curve implied the threshold of the lowest short-term mortality risk in ADHF patients. On the one hand, this threshold is significant for risk assessment, and on the other hand, maintaining the AIP around -0.1 in ADHF patients may be beneficial in improving their prognosis. Similar U-shaped associations for AIP have been reported in other diseases; in a study on the adverse prognosis of acute ischemic stroke patients (52), Liu H and colleagues found a U-shaped association between AIP quartiles (Q1-Q4) and 3-month mortality in stroke patients, with the lowest mortality risk associated with AIP values between -0.1 and 0.08. This finding is similar to our current study. Additionally, in a study by Lee MJ et al. (53), examining the relationship between AIP and all-cause mortality in dialysis patients, a U-shaped curve was observed, with the lowest mortality risk in dialysis patients around an AIP of 0.39. The AIP threshold reported by Lee MJ et al. differs from our findings, likely due to differences in study populations. It is well-known that renal impairment leads to changes in cholesterol structure, metabolism, reverse transport, accompanied by increased oxidative stress, electrolyte metabolism disorders, and other metabolic impacts, ultimately leading to dyslipidemia, particularly arteriosclerosis-inducing lipids (54–56). In our current analysis, we excluded patients with stage 5 chronic kidney disease and those undergoing dialysis to minimize the adverse impact of additional fluid and sodium retention on prognosis. Compared to Lee MJ et al.’s study on dialysis patients, our AIP levels were significantly lower [median value of the AIP: 0.075 (ours) vs 0.47 (Lee MJ et al.)].

The mechanism by which high AIP significantly increases the short-term mortality risk in ADHF patients is not entirely clear, but some insights may be provided by existing research evidence and auxiliary analyses in our study. Arteriosclerosis is known to be a pathological process involving long-term accumulation and transformation of lipids, inflammatory cells, smooth muscle cells, and necrotic cell debris beneath the endothelial cells lining the inner walls of arteries (57). Previously, arteriosclerosis was considered a lipid storage disease; however, more recent research has revealed it to be an ongoing inflammatory process (58, 59). Inflammation mediates the appearance of lipid streaks, the formation of arteriosclerosis, and subsequent cardiovascular and cerebrovascular complications, playing a pivotal role in the development of arteriosclerosis (58, 60). Fundamentally, arteriosclerosis can be termed an arteritis (57–60). Based on these established theories, our current study aimed to validate the role of inflammation in adverse cardiovascular outcomes associated with arteriosclerosis in real-world clinical practice. Using a mediation analysis model with WBC as the inflammatory marker (37), our results showed that inflammation significantly mediates the association between AIP and the 30-day mortality rate in ADHF patients, accounting for approximately 24.29% of the effect. This finding further validates the accuracy of basic research and quantifies the mediation effect of inflammation in this process, providing useful clinical data to support the basic mechanisms. Moreover, considering the significant impact of oxidative stress and nutrition on the pathogenesis and prognosis of ADHF patients (32, 34), we assessed the mediating effects of oxidative stress and nutrition. Our findings indicated that nutrition played a mediating role of about 8.16% in the association between AIP and 30-day mortality rate in ADHF patients, while the mediating effect of oxidative stress was not significant. Based on our findings, we recommend that for ADHF patients with high AIP, it is important to assess and manage inflammation and nutritional status, with potential benefits from enhanced nutritional support and anti-inflammatory treatment when necessary.

### Study strengths and limitations

Our study has several notable strengths: (i) This is the first report of the relationship between AIP and the prognosis of ADHF patients. (ii) The discovery of a U-shaped curve association is of significant clinical importance, as the AIP threshold indicating the lowest death risk can provide crucial assistance in risk assessment and treatment for ADHF patients. (iii) The findings from the mediation analysis offer a mechanistic explanation for the association between AIP and 30-day mortality risk in ADHF patients and also provide insights for future treatment directions.

However, our study also has certain limitations: (i) Being observational, it inevitably includes some unmeasured factors leading to residual confounding. Nevertheless, the calculated E-value (7.34) suggests that it is unlikely that any confounding factors could significantly alter our findings. (ii) We lack repeated measurements of AIP, which might be more beneficial for early risk stratification in ADHF patients. (iii) The study evidence is primarily applicable to the population in Jiangxi, and its relevance to other regions and ethnicities should be interpreted with caution. (iv) Due to the limited sample size, we did not observe significant associations in subgroups after further stratification. (v) The observational nature of the study limits our ability to further assess the impact of enhanced nutritional support and anti-inflammatory treatment on adverse outcomes in patients with high AIP. (vi) The causes of ADHF were not distinguished in the current study; considering the significant adverse cardiovascular effects of pre-existent cardiomyopathy, infections, ischemic heart disease, heavy alcohol use or illegal drug use and some chemotherapy medicines in patients with ADHF that were already present prior to the onset of the disease (61–65), this may result in some special populations being unobserved, and further studies are needed.

## Conclusion

In this retrospective cohort analysis, we have unveiled for the first time the association between AIP and the 30-day mortality rate in ADHF patients. Notably, this association exhibits a U-shaped curve before AIP<0.24, with the lowest 30-day mortality risk in ADHF patients around an AIP of -0.1. Additionally, based on evidence from mediation analysis, we have identified significant mediating effects of inflammation and nutrition on the association of AIP with the 30-day mortality rate in ADHF patients, with inflammation accounting for approximately 24.29% and nutrition for about 8.16% of the mediation effect.

## Data availability statement

The raw data supporting the conclusions of this article will be made available by the authors, without undue reservation.

## Ethics statement

The studies involving humans were approved by the Ethics Committee of Jiangxi Provincial People’s Hospital. The studies were conducted in accordance with the local legislation and institutional requirements. The participants provided their written informed consent to participate in this study.

## Author contributions

MY: Formal analysis, Investigation, Software, Validation, Writing – original draft. HY: Investigation, Writing – original draft. MK: Investigation, Software, Writing – review & editing. JQ: Investigation, Writing – review & editing. CY: Investigation, Writing – review & editing. GX: Data curation, Formal analysis, Investigation, Validation, Writing – review & editing. GS: Data curation, Formal analysis, Validation, Writing – review & editing. YZ: Conceptualization, Data curation, Formal analysis, Investigation, Methodology, Project administration, Software, Supervision, Validation, Writing – review & editing.
