# Supplementary material for: Atherogenic index of plasma: a new indicator for assessing the short-term mortality of patients with acute decompensated heart failure
Source: Front Endocrinol (Lausanne). 2024 Jun 10;15:1393644. doi: 10.3389/fendo.2024.1393644 (PMC11194402; doi:10.3389/fendo.2024.1393644)
Supplement: Supplementary file 1 [file DataSheet_1.docx]

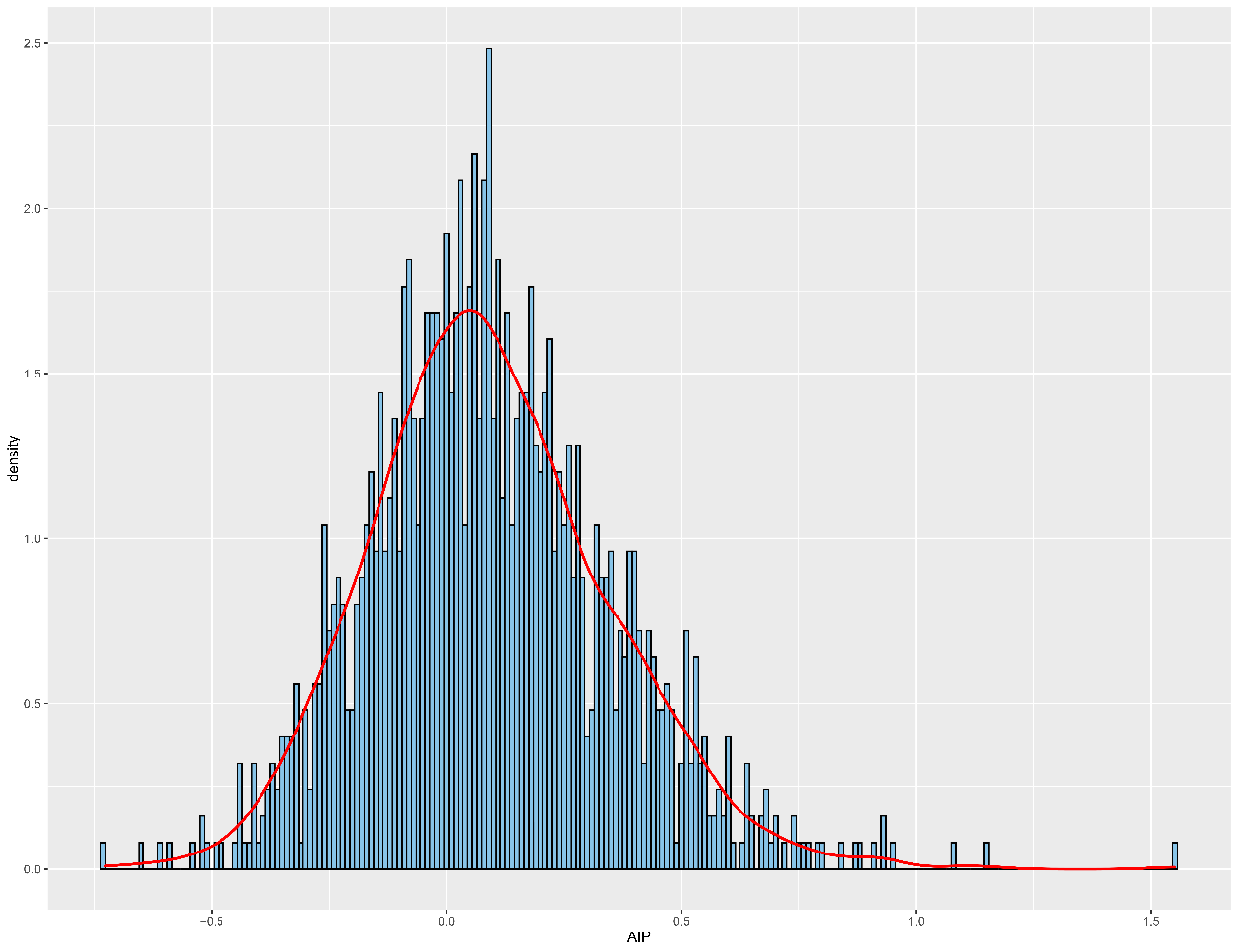


Supplementary Figure 1: Histograms show the population distribution of the AIP.


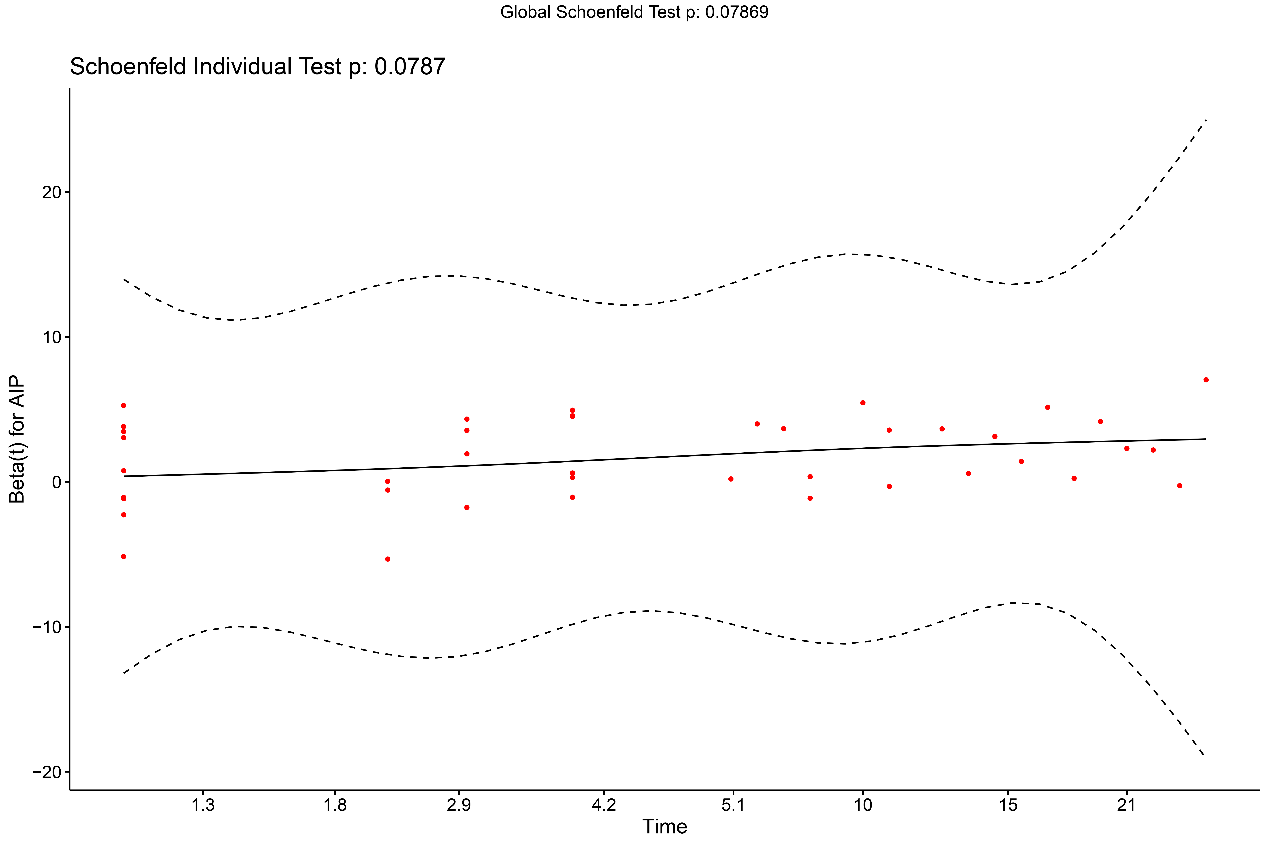


**Supplementary Figure 2**: Schoenfeld residual plot of AIP over time with 30-day mortality in ADHF patients as the dependent variable. The p-value of Schoenfeld Residuals Test result is larger than 0.05 which indicated that AIP is not a time dependent variable and can be analyzed by Cox Proportional Hazards Model.
