# Supplementary material for: Atherogenic index of plasma: a new indicator for assessing the short-term mortality of patients with acute decompensated heart failure
Source: Front Endocrinol (Lausanne). 2024 Jun 10;15:1393644. doi: 10.3389/fendo.2024.1393644 (PMC11194402; doi:10.3389/fendo.2024.1393644)
Supplement: Supplementary file 2 [file DataSheet_2.docx]

Supplementary Table 1: Collinearity diagnostics steps.

|  | VIF | | | |
| --- | --- | --- | --- | --- |
|  | Step 1 | Step 2 | Step 3 | Step 4 |
| AIP | 1.2 | 1.2 | 1.2 | 1.2 |
| Gender | 1.2 | 1.2 | 1.2 | 1.2 |
| Age | 1.4 | 1.4 | 1.4 | 1.4 |
| Hypertension | 1.3 | 1.3 | 1.3 | 1.3 |
| Diabetes | 1.2 | 1.2 | 1.2 | 1.2 |
| Cerebral infarction | 1.1 | 1.1 | 1.1 | 1.1 |
| CHD | 1.2 | 1.2 | 1.2 | 1.2 |
| NYHA classification | 1.1 | 1.1 | 1.1 | 1.1 |
| SBP | 1.8 | 1.8 | 1.8 | 1.8 |
| DBP | 1.5 | 1.5 | 1.5 | 1.5 |
| LVEF | 1.2 | 1.2 | 1.2 | 1.2 |
| WBC | 1.4 | 1.4 | 1.4 | 1.4 |
| RBC | 4.1 | 4.1 | 4.1 | 1.4 |
| HGB | 4.3 | 4.3 | 4.3 | NA |
| PLT | 1.3 | 1.3 | 1.3 | 1.3 |
| ALB | 1.3 | 1.3 | 1.3 | 1.2 |
| ALT | 7.7 | NA | NA | NA |
| AST | 7.5 | 1.2 | 1.2 | 1.2 |
| GGT | 1.1 | 1.1 | 1.1 | 1.1 |
| Cr | 1.5 | 1.5 | 1.5 | 1.5 |
| UA | 1.4 | 1.4 | 1.4 | 1.4 |
| TC | 4.7 | 4.7 | NA | NA |
| LDL-C | 4.5 | 4.5 | 1.2 | 1.1 |
| NT-proBNP | 1.2 | 1.2 | 1.2 | 1.2 |

VIF: variance inflation factor; VIF = 1/(1-R^2^). Abbreviations as in Table ​1.

Note: The variables with VIF>3 will be regarded as collinear variables and cannot be included in the multiple regression model.
